# Metabolic Characterization of a Novel RORα Knockout Mouse Model without Ataxia

**DOI:** 10.3389/fendo.2017.00141

**Published:** 2017-07-11

**Authors:** Cyrielle Billon, Sadichha Sitaula, Thomas P. Burris

**Affiliations:** ^1^Department of Pharmacology and Physiology, Saint Louis University School of Medicine, St. Louis, MO, United States

**Keywords:** lipids, carbohydrates, nuclear receptor, metabolism, metabolic disease

## Abstract

The retinoic acid receptor-related receptor α (RORα) is a nuclear receptor that plays an important role in regulation of metabolism and the immune system. Genetic deletion of the receptor yields mice with significant cerebellar developmental issues associated with severe ataxia. Although many metabolic studies have been performed in these models, the impaired locomotor activity of these mice is known to affect their normal mobility and feeding behaviors. This creates some difficulty in interpretation of the role of RORα in models of metabolic disease where feeding and muscle function is a critical component of the pathophysiology. We generated a mouse with a floxed *Rora* allele that we crossed with a mouse line expressing Cre recombinase under the control of the EIIa promoter to obtain a full body deletion of *Rora*. This cross led to a partial deletion of the *Rora* locus likely due to mosaic expression of the EIIa-Cre transgene. These mice lack any signs of ataxia but display an improved metabolic profile relative to normal WT mice. The mice were resistant to diet- and age-induced metabolic syndrome and exhibited improved glucose tolerance and increased insulin sensitivity. Decreased RORα expression in the mice was also associated with reduced inflammation in models of metabolic syndrome. These data indicate that suppression of RORα activity improves metabolic function and reduces inflammation.

## Introduction

More than two-thirds of American adults are considered to be overweight or obese. Obesity is associated with a range of diseases including diabetes, cardiovascular diseases and several cancers. Obesity is the result of a positive energy balance, a consequence of excess caloric intake relative to calories expended. Energy expenditure is the combination of resting metabolic rate (energy consumed at rest), the thermogenic effect of food (energy utilized to metabolize food consumed), and energy expended during physical activity. When energy expenditure exceeds energy intake, it results in a negative energy balance leading to weight loss. The mechanisms underlying the regulation of energy balance are not completely understood, but evidence suggests that complex physiological regulation and genetic predisposition are involved (1). In addition to behavioral interventions, such as dieting and exercise, various pharmacological strategies are also being examined to reduce obesity focused on reducing caloric intake as well as increasing energy expenditure.

Nuclear receptors (NRs) are ligand-dependent transcription factors that regulate many biological functions including development, metabolism, and inflammation (2). In the context of metabolism, NRs control lipid and glucose homeostasis in a tissue-specific manner. Dysfunction of NR signaling has been shown to be associated with dyslipidemia, insulin resistance, diabetes, obesity, and cardiovascular diseases. Retinoic acid receptor-related orphan receptors (RORs) represent a subfamily of the NRs and are involved in regulation of metabolism and inflammation as well as the pathogenesis of atherosclerosis (3–5). The ROR family is composed of three members: RORα, RORβ, and RORγ. Both RORα and RORγ are expressed in immune cells as well as other tissues known to be the key in regulation of metabolic processes in the liver and skeletal muscle (6). Several studies have implicated RORα in regulation of glucose and lipid metabolism as well as inflammatory and immune responses *in vivo* using the staggerer (RORα^sg/sg^) mouse model (7, 8). In staggerer mice, the gene encoding RORα contains a 6.5 kb genomic deletion leading to loss of an exon that encodes part of its ligand-binding domain (LBD). This leads to an exon skip that shifts the reading frame and introduces a premature stop codon, providing a truncated protein lacking the entire LBD that is believed to function as a dominant negative protein. Unfortunately, these mice develop severe ataxia (hence the name “staggerer”) due to a deficit in Purkinje cell development that impairs mobility and normal feeding behavior, making the study of metabolism more complex in this model. The RORα null mice display an identical phenotype recapitulating the severe ataxia (9).

In this study, we describe a new mouse model of deletion of RORα that does not display ataxia providing an improved model for assessing RORα-dependent metabolism where “normal” feeding behavior and locomotion are retained. We generated a mouse with a floxed *Rora* allele that we crossed with a mouse line expressing Cre recombinase under the control of the EIIa promoter to obtain a full body deletion of *Rora*. As the EIIa-cre model induced a mosaic expression of the cre recombinase, we did not obtain a total recombination of the *Rora* Flox loci but a partial deletion. These animals (EIIaRORα KO) lack any signs of ataxia but display an improved metabolic profile relative to normal EIIaRORα WT mice. Our study presents a new model to study the role of RORα in metabolic disorders without the complexity of a cerebellar deficit that may alter normal feeding behavior and physical activity. As RORα can be targeted by synthetic ligands (4, 10), these data suggest that pharmacological modulation of RORα may be useful to treat metabolic syndrome and related diseases such as diabetes and liver diseases.

## Materials and Methods

### Animals

All procedures were approved and conducted in accordance to the St. Louis University Institutional Animal Care and Use Committee.

All mice were housed in a 12-h light/dark cycle. At the end of the experiment, mice were euthanized by CO_2_ asphyxiation followed by cervical dislocation. Body composition was analyzed weekly by NMR using BioSpin LF50 Body Composition Analyzer (Bruker, Germany). Tissues were collected, snap frozen and stored at −80°C for qPCR or western blotting, or fixed in 4% formalin, incubated in 20% sucrose overnight before freezing for cryosection.

### Gene Targeting and Generation of *Rora* Floxed Mice

**Figure 1:**
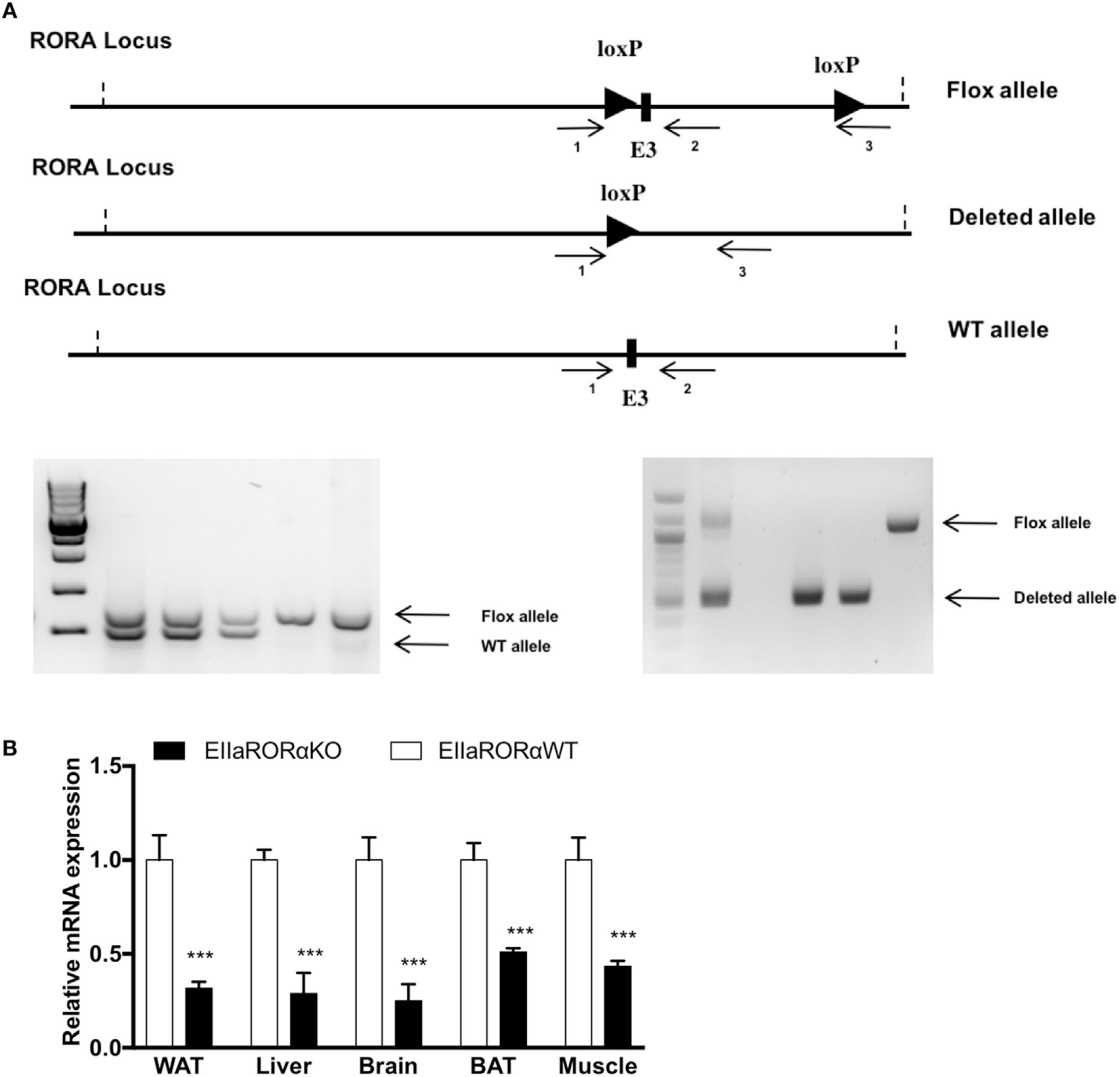
RORα flox loci construct and validation of RORα deletion. **(A)** Schematic representation of the LoxP sequence in RORα loci and primer localization. To assess the presence of the LoxP sites and the deleted allele after action of the cre recombinase, two sets of primers were designed (1–2 and 1–3). 1.5%-Agarose gel representing the genotyping results after PCR amplification. The WT and Flox alleles were amplified with the set of primer 1–3, and the deleted allele was amplified with the set of primers 1–3. **(B)** White adipose tissue (WAT), liver, brain, brown adipose tissue (BAT), and muscle gene expression for RORα from 2-month-old EIIARORα WT (white bar, *n* = 6) and EIIARORα KO (black bar, *n* = 6) (***p < 0.001).

The floxed RORα mouse was created by the transgenics core at the Pennington Biomedical Research Center (Baton Rouge, LA, USA) by inserting LoxP sites by homologous recombination flanking the exon three of the *rora* gene. Female *Rora*^flox/flox^ mice were bred with male mice homozygous for the Cre recombinase gene under the control of the adenovirus EIIA promoter to generate the *Rora*^+^/ΔCre F1 generation. EIIa-Cre transgenic mice were obtained from The Jackson Laboratory (Bar Harbor, ME, USA; stock number: 003724). Genotypes were determined by PCR using the oligonucleotide primers: primer 1: 5′-GCCCCCTTTCCCGCCAGTAGCTG-3′; primer 2: 5′-GACCCAGATCCCCTAATAACG-3′; primer 3: 5′-GCCCTCAAGAAGAGGCTGCAATTT-3′ (Figure 1A).

### Indirect Calorimetry

Respiratory exchange ratio (RER), VO_2_, VCO_2_, heat production, and activity were measured using a monitoring system (Oxymax Comprehensive Lab Animal System; Columbus Instruments, Columbus, OH, USA). Eight aged-matched male mice (EIIaRORα WT and EIIaRORα KO) were housed individually in metabolic cages on a 12 h day–night cycle, fed with either a normal chow or high fat diet (HFD) (D12492, Research diet). After a 24-h acclimatization period, data were collected every 16 min over a 5-day period. The hourly or average values during light and dark periods were calculated. Two-way ANOVA followed by Bonferroni posttest was used to calculate the *p* value.

### Glucose and Insulin Tolerance Test (ITT)

After 6-h fast, EIIaRORα WT and EIIaRORα KO (*n* = 8) were injected intraperitoneally with glucose (2 g/kg of fat free mass) or insulin (0.75 U/kg of fat free mass) (Sigma-Aldrich, St. Louis, MO, USA) to examine glucose tolerance test or ITT, respectively. Blood glucose was measured before the injection (*t* = 0 min), 15, 30, 60, and 120 min after injection using OneTouch Ultra^®^2 glucometer. Blood was collected by tail snip; the first drop was wiped out, and the second drop was used to quantify glucose level.

### Plasma Lipid, Liver Enzyme, and Cytokines Analysis

Mice were euthanized, and blood was collected *via* cardiac puncture. Concentration of plasma total cholesterol, high-density lipoprotein (HDL) cholesterol, LDL cholesterol, triglyceride, and liver enzymes were assessed using Rx Daytona clinical chemistry analyzer (Randox). Plasma TNFα or IL-1β concentrations were detected by ELISA (Mouse TNF alpha ELISA KIT, EMD Millipore and Mouse IL-1 beta Quantikine SixPak, R&D Systems, respectively) according to the manufacturer’s recommendations.

### Quantitative Real-time PCR

Total RNA was isolated from mouse tissues using PureLink RNA mini kit (Ambion, Life Technologies). RNA was reverse transcribed to make cDNA using qScript™ cDNA Synthesis Kit (Quanta biosciences) according to the manufacturer’s instructions. Real-time PCR was performed using a SYBR-green PCR master mix kit (SYBR SELECT MASTER MIX, Life Technologies). Primers were purchased from Integrated DNA Technologies (see Table S1 in Supplementary Material for sequences).

### Flow Cytometry

Spleens and lymph nodes were harvested into a tissue culture dish and teased apart into a single cell suspension. Cell suspension was passed through a cell 0.22 µM filter (EMD Millipore) and centrifuged (300–400 × *g*) at 4°C. Cell pellet was resuspended in Flow Cytometry Staining Buffer (BioLegend, San Diego, CA, USA) at the final concentration of 2 × 10^7^ cells/mL. Cell surface and intracellular staining were performed according BioLegend protocol (BioLegend). Antibodies used for analysis are listed in Table S2 in Supplementary Material.

### Statistical Analysis

Data are expressed as mean ± SEM. Student’s *t*-test or two-way ANOVA was used to calculate statistical significance. *p* < 0.05 was considered significant.

## Results

### EIIaRORα KO Mice Do Not Display a Staggerer Phenotype

Previous studies have shown that homozygous staggerer mice are leaner and display improved insulin sensitivity but also have a severe cerebellar ataxia (9). To help normalize feeding, these ataxic mice are typically provided food on the bottom of the cage so as to provide more ready access given their locomotor deficits (9). We generated mice heterozygous for a transgene encoding Cre recombinase expressed under the direction of the EIIa promoter (EIIa-Cre), which directs expression in all tissues, and homozygous for *lox*P-flanked exon 3 of *Rora* gene [Figure 1A, *Rora*^flox/flox^Cre^+/WT^ (EIIaRORα KO)]. *Rora*^flox/flox^ littermates without the EIIa-Cre transgene (EIIaRORα WT) served as controls. When we assessed the level of *Rora* expression in different tissues, we found that the deletion was not complete. *Rora* expression ranged from 45 to 30% of normal levels depending on the tissue examined. We observed a significant decrease of expression in several tissues: 65% in white adipose tissue (WAT), 65% in liver, 70% in brain, 55% in BAT, and 60% in the skeletal muscle (Figure 1B). The EllaRORα KO mice displayed normal locomotion (Figure S1 in Supplementary Material, activity data at 3-month old) and exhibited no signs of ataxia, from weaning time (day 21) to 15 months of age (data not shown). The residual RORα expression in the brain may explain why the EIIaRORα KO mice do not display ataxia. Overall, no differences in the size or the weight of several organs (liver, kidney, spleen, quadriceps, brown or WATs, heart) were observed (data not shown). There were no gross alterations in brain morphology, but fine characterization of brain morphology and function will be examined in later studies.

### EIIaRORα KO Mice Preferentially Use Fat for Energy

**Figure 2:**
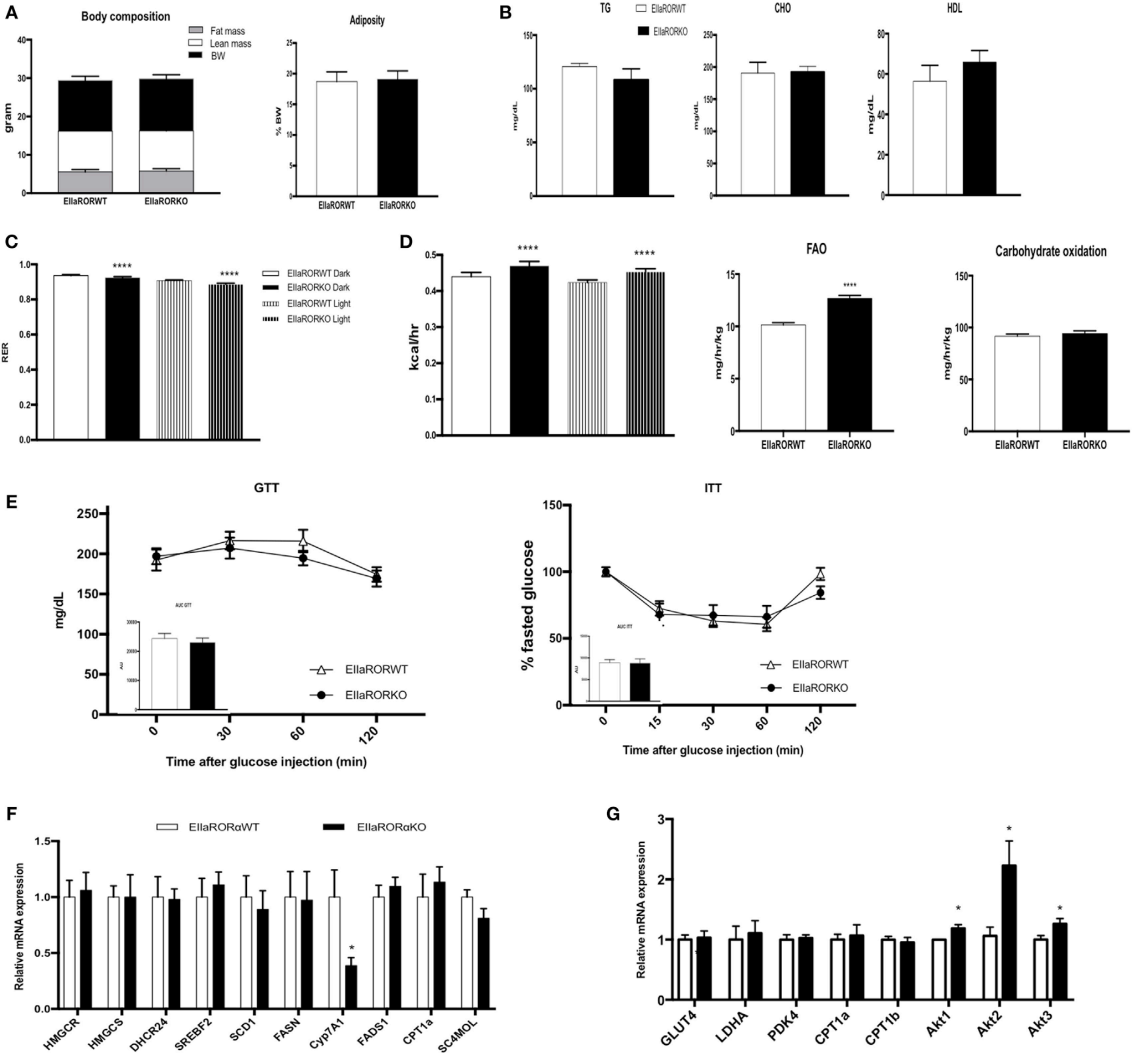
EIIARORα KO mice display an increase fatty acid oxidation and energy expenditure. **(A)** Body composition of single-housed 12-week-old males EIIARORα WT (*n* = 8) and EIIARORα KO (*n* = 8) littermates (left panel) and adiposity of the same animals (right panel) fed with a normal chow diet. **(B)** Blood lipid profile (triglycerides, total cholesterol, and HLD) of the same males EIIARORα WT (white bar, *n* = 8) and EIIARORα KO (black bar, *n* = 8). **(C)** Respiration exchange ratio and **(D)** heat production of the same EIIARORα WT (white, *n* = 8) and EIIARORα KO (black, *n* = 8) littermates during day (solid pattern) or night (shaded pattern) over a 5-day period and after 24 h acclimation (upper panel). Fatty acid oxidation and carbohydrates consumption of the same males EIIARORα WT (white bar, *n* = 8) and EIIARORα KO (black bar, *n* = 8) calculated over a 5-day period. **(E)** Glucose (left) and insulin (right) tolerance test from the same males EIIARORα WT (white triangle, *n* = 8) and EIIARORα KO (black square, *n* = 8). Area under the curve is represented on each graph. **(F)** Liver and **(G)** muscle gene expression from the same males EIIARORα WT (white bar, *n* = 8) and EIIARORα KO (black bar, *n* = 8) (*p < 0.05, ****p < 0.0001).

Three-month-old male mice (EIIaRORα KO or EIIaRORα WT) were fed with a chow diet and used to decipher the role of RORα in energy and glucose metabolism. Whole body composition was determined weekly using NMR (BioSpin LF50, Bruker Germany), and no significant differences were observed in body weight, lean mass, or fat mass (Figure 2A). Using indirect calorimetry, we were able to determine that deletion of RORα leads to an increase in fat oxidation during both day- and nighttime (Figures 2C,D). No changes in carbohydrate consumption were observed, but we observed an increase in the total quantity of fat oxidized. This increase of fat utilization led to an increase in energy expenditure with neither an increase in food intake nor activity (Figure S1 in Supplementary Material). Typically, an RER of 0.7 indicates that mice are fasted and a difference of 0.05 in the RER reflects a change of 20% of fuel utilization. Fat oxidation was increased by 20% in EIIaRORα KO compared to EIIaRORα WT mice when fed a normal chow diet. No differences were observed in levels of blood triglycerides, total cholesterol, HDL, or blood glucose in fasted or fed mice (Figure 2B). RORα deletion did not alter glucose or insulin tolerance (Figure 2E). Expression of several lipogenic and cholesterologenic enzyme genes in liver was also unaffected by RORα deletion (Figure 2F). Interestingly, Akt signaling was affected by RORα deletion. All Akt isoforms (Akt1, 2, and 3) were upregulated in EIIaRORα KO compared to EIIaRORα WT mice (Figure 2G). These results demonstrate that RORα deletion leads to an increase in fat utilization when mice are fed a normal chow diet, but loss of RORα has no effect on glucose utilization or hepatic cholesterol synthesis.

### EIIaRORα KO Mice Are Resistant to Diet-Induced Obesity (DIO)

**Figure 3:**
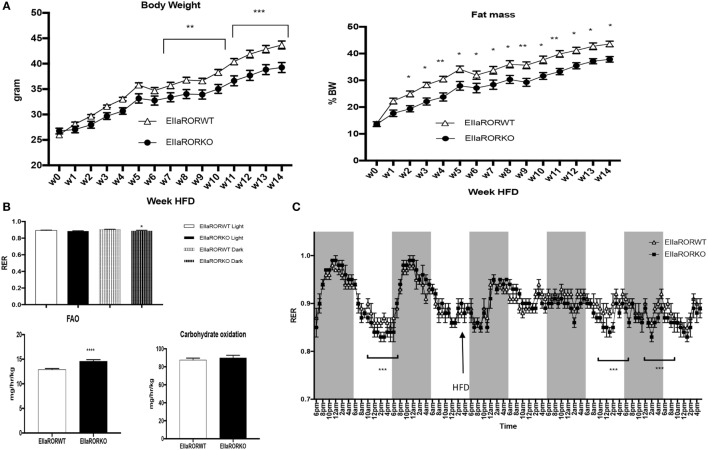
EIIARORα KO mice are resistant to diet-induced obesity. **(A)** Weekly body weight and adiposity of 3-month-old males EIIARORα WT (white triangle, *n* = 7) and EIIARORα KO (black square, *n* = 8) mice fed with high fat diet (HFD). **(B)** Fatty acid oxidation (left) and carbohydrates consumption (right) of the same males EIIARORα WT (white bar, *n* = 7) and EIIARORα KO (black bar, *n* = 8) fed with HFD calculated over a 5-day period (*p < 0.05, **p < 0.01, ***p < 0.001). **(C)** Indirect calorimetry measurement of the same animals EIIARORα WT (white triangle, *n* = 7) and EIIARORα KO (black square, *n* = 8) under chow diet and then challenged (black arrow) with HFD (right panel). Average of respiratory exchange ratio (RER) value for day and nighttime of the same EIIARORα WT (white, *n* = 7) and EIIARORα KO (black, *n* = 8) littermates during day (solid pattern) or night (shaded pattern) over a 5-day period under HFD.

Three-month-old male mice were challenged with an HFD (60% calories from fat, Harland TD06414) over 14 weeks and monitored for body composition weekly, fuel utilization (at week 1 of HFD) and glucose metabolism. EIIaRORα KO mice exhibited resistance to DIO displaying significantly reduced body weight gain and reduced fat mass relative to control mice (Figure 3A). After 7 weeks on an HFD, a significant difference in body weight was observed (Figure 3A, left panel) and, surprisingly, after only 2 weeks on an HFD, fat mass was lower in EIIaRORα KO mice compared to EIIaRORα WT animals (Figure 3A, right panel). Three-month-old mice were maintained in the metabolic chambers for 2 days on a regular chow diet and then challenged with the same HFD. Metabolic parameters such as RER, oxygen consumption, activity, and energy expenditure were monitored for 5 days. After transition to the HFD, the RER displayed a less pronounced circadian pattern of oscillations (Figure 3B). During both day- and nighttime, the RER of EIIaRORα KO animals was lower than the wild-type counterparts (Figure 3B) consistent with an increase in fatty acid oxidation in the KO mice. We calculated the amount of fatty acid oxidized per hour per kilogram of mouse using the Frayn’s equation (11). We observed 13.5% increase in the quantity of fatty acid oxidized in the EIIaRORα KO animals compared to control mice (Figure 3C, right panel). No change in carbohydrate oxidation was observed (Figure 3C, left panel). These data suggest that EIIARORα KO mice were resistant to DIO due to an increase of fatty acid oxidation even with food intake remaining constant (Figure S1 in Supplementary Material).

### HFD-Fed RORα KO Mice Display Improved Glucose Metabolism and an Anti-inflammatory Profile

**Figure 4:**
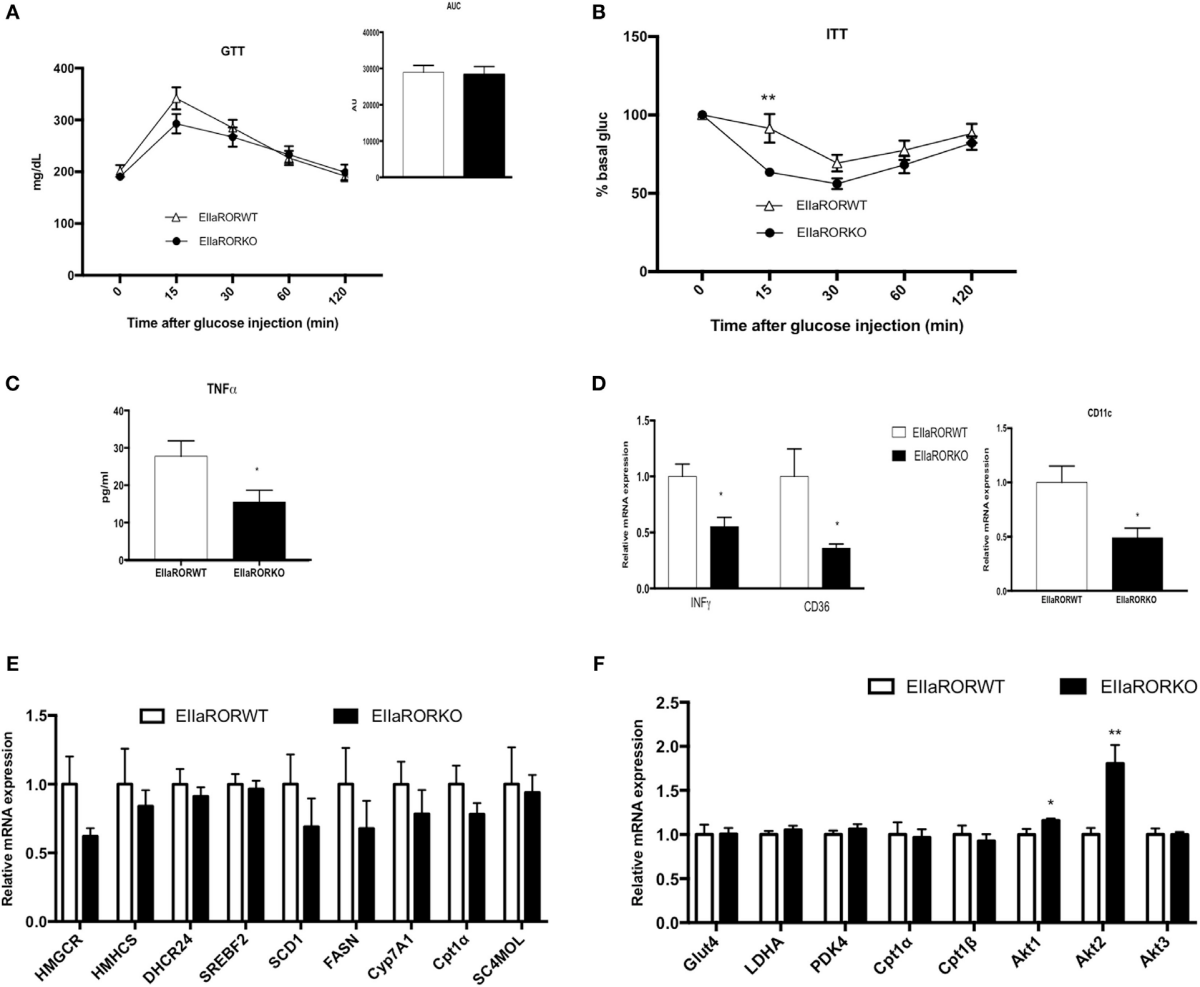
EIIARORα KO mice fed with high fat diet (HFD) display a better glucose homeostasis than EIIARORα WT mice. **(A)** Glucose and **(B)** insulin tolerance test from the same males EIIARORα WT (white triangle, *n* = 7) and EIIARORα KO (black square, *n* = 7) used in the Figure 3, fed with HFD for 8 and 9 weeks, respectively. Area under the curve is represented on each graph. **(C)** Blood TNFα level of the same males EIIARORα WT (white triangle, *n* = 7) and EIIARORα KO (black square, *n* = 7) fed with HFD for 14 weeks quantified by ELISA. **(D)** WAT gene expression from the same males EIIARORα WT (white triangle, *n* = 7) and EIIARORα KO (black square, *n* = 7) fed with HFD for 14 weeks. **(E)** Liver and **(F)** muscle gene expression from the same males EIIARORα WT (white bar, *n* = 7) and EIIARORα KO (black bar, *n* = 7) (*p < 0.05, **p < 0.01).

One side effect of obesity is impaired glucose metabolism, which is associated with insulin resistance and type-2 diabetes. We assessed the effect of RORα deletion in glucose metabolism in HFD-fed mice for 8 weeks. At this time point, a significant difference in body weight and adiposity was observed between EIIARORα KO mice and their control littermates (Figure 3A). As no differences were observed in lean mass and to avoid an effect due to the body weight, we decided to normalize the amount of glucose or insulin injected to lean mass. No difference in response to glucose (Figure 4A) was observed between the genotypes, but EIIARORα KO mice were more sensitive to insulin compared to their EIIARORα WT littermates (Figure 4B). No differences were observed in fasted blood glucose levels between EIIARORα KO animals and their control littermates (Figure S2 in Supplementary Material). As obesity is also considered an inflammatory disease, we also assessed the blood levels of pro-inflammatory cytokines IL-1β and TNFα. No differences were observed between the two genotypes with respect to circulating IL-1β level, but we found a significant decrease in circulating TNFα levels in EIIARORα KO animals compared to EIIARORα WT mice (Figure 4B; Figure S2 in Supplementary Material). As the WAT can be a source of inflammation, we quantified inflammatory gene expression in this tissue. Most of the pro-inflammatory markers (TNFα, IFNγ, CD36, CD11c) displayed a lower level of expression in EIIARORα KO animals compared to EIIARORα WT mice (Figures 4C,D; Figure S2 in Supplementary Material). T lymphocytes also play a critical role in WAT-associated inflammation and gene expression for FoxP3, a marker for regulatory T cells (T_reg_), was increased in EIIARORα KO animals compared to EIIARORα WT suggesting more infiltration of T_reg_ cells (Figure S2 in Supplementary Material). Previously, we demonstrated that EIIARORα KO mice display an anti-inflammatory profile with a decrease in CD4^+^ and CD8^+^ cell populations in the spleen when fed a chow diet (5). These results show that EIIARORα KO mice display a lower level of inflammation and improved glucose metabolism relative to EIIARORα WT mice when they are challenged with an HFD. The expression of several genes encoding lipogenic enzymes was analyzed by qPCR, but no differences were observed between EIIARORα WT and EIIARORα KO animals (Figure 4E). Since EIIARORα KO mice are more sensitive to insulin, we assessed the role of RORα deletion in skeletal muscle. The expression of several genes involved in glucose and insulin metabolism was assessed by qPCR, and no major differences were observed between EIIARORα WT and EIIARORα KO except for Akt expression (Figure 4F).

### Aged-EIIARORα KO Mice Display Improved Glucose Metabolism and an Anti-inflammatory Profile

**Figure 5:**
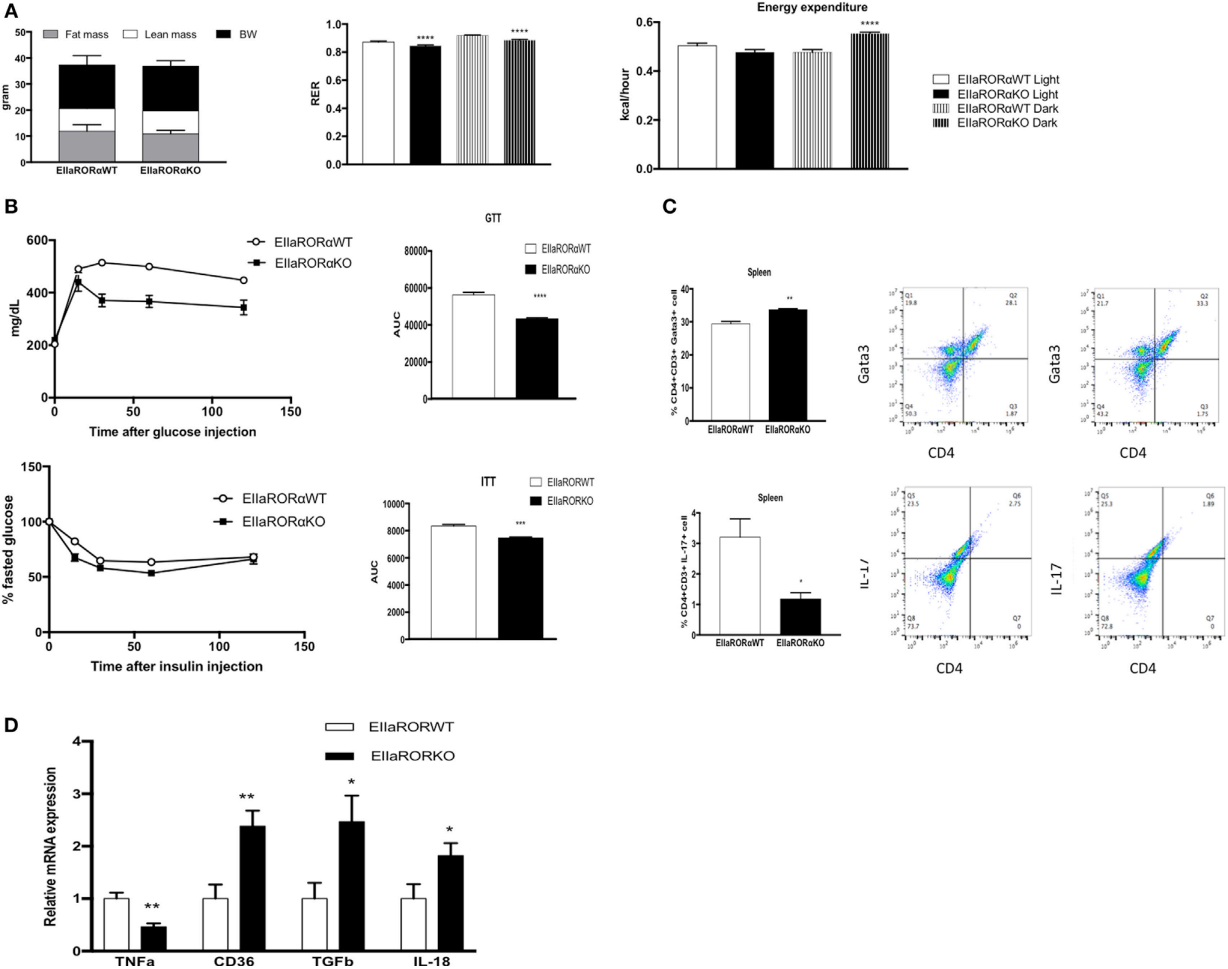
Aged-EIIARORα KO display improved glucose homeostasis and a lower inflammation rate than EIIARORα WT mice. **(A)** Body composition of single-housed 15-month-old males EIIARORα WT (*n* = 6) and EIIARORα KO (*n* = 7) littermates (left panel) fed with a normal chow diet. Respiration exchange ratio (middle panel) and heat production (right panel) of the same aged-EIIARORα WT (white, *n* = 6) and EIIARORα KO (black, *n* = 7) littermates during day (solid pattern) or night (shaded pattern) over a 5-day period and after 24 h acclimation (upper panel). **(B)** Glucose (upper panel) and insulin (lower panel) tolerance test from the same males aged-EIIARORα WT (white triangle, *n* = 6) and EIIARORα KO (black square, *n* = 7) fed with a normal chow diet done at 13-month old. Area under the curve is represented on each graph. **(C)** Splenocytes from 15-month-old males EIIARORα WT (white bar, *n* = 6) and EIIARORα KO (black bar, *n* = 7) littermates were analyzed by flow cytometry for T_H_2 [CD3^+^CD4^+^Gata3^+^ (upper panel)], or T_H_17 [CD3^+^CD4^+^IL-17^+^ (lower panel)]. **(D)** WAT gene expression from the same 15-month-old males EIIARORα WT (white bar, *n* = 6) and EIIARORα KO (black bar, *n* = 7) littermates (*p < 0.05, **p < 0.01, ***p < 0.001, ****p < 0.0001).

Risk for development of metabolic disorders increases with age, thus we decided to study the effect of RORα deletion in aging. We used 15-month-old male EIIARORα KO and EIIARORα WT mice maintained on a normal chow diet. No differences in body composition were observed between the two groups (Figure 5A). To determine whether old-EIIARORα KO mice exhibited increased energy expenditure, we measured activity, heat production, as well as the RER over a 5-day period. No changes were observed in activity, but we observed a decrease in RER suggesting an increase in fat utilization during the resting period (day time) as well as an increase in heat production (Figure 5A). These data suggest that EIIARORα KO mice have a higher rate of energy expenditure and fatty acid oxidation. We also observed that old-EIIARORα KO mice were more glucose tolerant than old-EIIARORα WT mice (Figure 5B). When we challenged these mice with insulin, old-EIIARORα KO mice were more sensitive than the WT mice (Figure 5B). No differences were observed in fasted blood glucose levels (Figure S3 in Supplementary Material). Aging is also associated with increased pro-inflammatory cells and cytokines, thus we studied the proportion of different lymphocytes cell populations in the spleens of these mice by flow cytometry. An increase in anti-inflammatory T_H_2 lymphocytes (CD4^+^CD3^+^Gata3^+^) was observed in old-EIIARORα KO mice compared to old-EIIARORα WT mice as well as a decrease of pro-inflammatory lymphocytes T_H_17 (CD4^+^CD3^+^IL-17^+^; Figure 5C). No changes were observed in number of T_H_1 lymphocytes or T_reg_ lymphocytes between the genotypes (Figure S3 in Supplementary Material). In WAT, a decrease in TNFα expression and an increase the expression of anti-inflammatory genes such as TGFβ and macrophage infiltration marker CD36 were observed in old-EIIARORα KO mice compared to old-EIIARORα WT mice (Figure 5D). These results suggest that deletion of RORα can be protective in age-induced metabolic disorders such as diabetes and obesity.

## Discussion

In this study, we have described a novel mouse model that we used to characterize the role of RORα in metabolic disorders. These EIIARORα KO mice, which expressed significantly reduced RORα expression relative to WT mice but did not display mobility/ataxia issues typically associated with loss of RORα function, were protected against diet- and age-induced metabolic syndrome. In these mice, reduction of RORα expression protected against DIO and improved glucose metabolism and insulin resistance were observed while also decreasing markers of inflammation. Our study extends previous ones utilizing the staggerer mouse model that also suggest a metabolic benefic of RORα deletion (7, 8). However, metabolic studies in the staggerer mouse have typically been difficult to interpret due to significant developmental abnormalities in these mice leading to cerebellar ataxia. Interestingly, our new mouse model that exhibits a partial deletion of RORα lacks the troublesome ataxia that leads to abnormal movement and feeding, but we were still able to clearly link lower RORα activity to improved glucose metabolism, reduced insulin resistance, resistance to diet- or age-induced obesity, and reduced inflammation. These mice also display increased energy expenditure and increased fatty acid oxidation and a reduction in fat mass when maintained on a normal chow diet or an HF diet. Obesity is associated with mild systemic inflammation and that inflammation plays a critical role in pathologies associated to obesity like cardiovascular diseases as well as diabetes. Our study has shown a correlation between level of expression of RORα, inflammation, and resistance to diet- and age-induced obesity. Infiltration of immune cells, macrophages and T lymphocytes, in WAT leads to production of systemic pro-inflammatory cytokine that trigger the development of systemic inflammation found in obese patients or animal models (12–14). RORs (RORα and RORγ) are known to play a role in atherosclerosis and lymphocytes T_H_17 differentiation (4, 5). Our data have shown that a partial deletion of RORα is sufficient to reduce inflammation and induce T_reg_ differentiation and/or proliferation to prevent an increase in inflammation in the WAT. The spleen plays a major role in T cell maturation and activation, and our cytometry study shows an anti-inflammatory profile of T cells in aged-EIIARORα KO mice. Previously, we showed a similar profile in young EIIARORα KO mice under chow diet (5). All these data support the pro-inflammatory role of RORα, and modulation of its activity can protect against pathological inflammation. One of the key factors controlling T cell fate is the transcription factor hypoxia-inducible factor-1α (HIF-1α). HIF-1α is activated by the level of oxygen and can be regulated by the NF-κB signaling pathway in T cells. Activation of HIF-1α will induced a metabolic switch in cells, which dramatically influences T cell lineage commitment (15). HIF-1α can attenuate T_reg_ differentiation toward T_H_17. Interestingly, HIF-1α has been shown to by directly regulated by RORα (16). We hypothesized that the deletion of RORα can lead to a decrease of HIF-1α expression in naïve T cells (CD4^+^) that will promote T_reg_ differentiation and block T_H_17 differentiation.

Aged-EIIARORα KO or EIIARORα KO mice under HF diet for weeks are still insulin sensitive and glucose tolerant, suggesting that these mice are less susceptible to aged or diet-induced type-2 diabetes. Very interestingly, we observed an upregulation of Akt signaling in the muscle of EIIARORα KO animals, which could explain the more efficient insulin signaling. Further studies regarding the insulin pathway will be necessary to understand the role of RORα deletion in this process. The mechanisms underlying these results are not clear, but it could be a link to a decrease of inflammation and a better peripheral glucose metabolism. The muscles play a critical role in glucose metabolism and insulin sensitivity (17). Previous data obtained in the lab have shown that targeting RORα and RORγ with synthetic inverse agonist reduces diabetes in a non-obese mouse model (18). The role of each ROR isotype is poorly understood in these mechanisms. Our model will allow us to also study the role of RORα in specific tissues such as muscle and pancreas and its impact in glucose metabolism and insulin sensitivity.

Obesity and fat accumulation are the results of imbalance between energy intake and expenditure. Indirect calorimetry analysis of 2-month-old EIIARORα KO and EIIARORα WT fed with normal chow diet as well as aged EIIARORα KO and EIIARORα WT have shown an increase in energy expenditure and fatty acid oxidation without alteration of food intake during both night- and daytime. We hypothesized that the increase in energy expenditure was linked to an increase in thermogenesis in the brown adipose tissue (BAT). To test this, we challenged young EIIARORα KO and EIIARORα WT to cold temperature, but no differences were observed in energy expenditure, fatty acid oxidation, or BAT activity (data not shown). We then challenged EIIARORα KO and EIIARORα WT mice with HF diet for 8 weeks before performing the same indirect calorimetry analysis. As EIIARORα KO mice accumulate less fat, the normalization to the fat free mass excludes any weight effect in energy expenditure. EIIARORα KO mice are utilizing more energy during both day- and nighttime and have an increase in fatty acid oxidation. Fatty acid oxidation has been shown to be impaired in aging animals (19), but our aged-EIIARORα KO still display an increase in fatty acid oxidation. These results lead to the hypothesis that RORα may regulate fatty acid oxidation and prevent against aged-dependent decrease of metabolism. The elevated energy expenditure and fatty acid oxidation observed in all our models might explain the reduced fat accumulation and insulin resistance in EIIARORα KO mice.

In conclusion, we developed a new RORα deficiency mouse model that recapitulates the observations performed in the Staggerer mice but without the problematic effects of ataxia. Partial deletion of RORα protects against diet- and age-induced metabolic syndromes and inflammation. This RORα floxed model will provide a new tool to study the role of RORα in metabolic syndrome. By generating tissue-specific RORα deletions using this model, we will be able to better understand the role of RORα in metabolic tissue and some of the problematic cerebellar developmental effects. We previously published that ROR can be targeted by synthetic ligands and protects against inflammation, diabetes, and cardiovascular diseases (4, 5, 18), but unfortunately, we have not been able to decipher the specific role of each receptor isotype nor the role of various isotypes within specific tissues with great precision. With this model, we will be able to better understand the role of RORα versus RORγ in these diseases and utilize this information to develop improved therapeutic strategies to manage and prevent obesity and related pathologies such as cardiovascular diseases and diabetes.

## Ethics Statement

All animal studies were approved by the SLU IACUC.

## Author Contributions

TB and CB conceptualized the project; wrote and edited the paper. CB and SS performed experiments. All the authors analyzed the data.

## Conflict of Interest Statement

The authors declare that the research was conducted in the absence of any commercial or financial relationships that could be construed as a potential conflict of interest.

## Supplementary Material

Figure S1 Food intake and activity of single-housed EIIARORα WT and EIIARORα KO mice. **(A)** Food intake of single-housed 12-week-old males EIIARORα WT (white bar, *n* = 8) and EIIARORα KO (black bar, *n* = 8) littermates under chow diet over a month. **(B)** Recording of single-housed 12-week-old males EIIARORα WT (white triangle, *n* = 8) and EIIARORα KO (black square, *n* = 8) littermate activity in metabolic chambers over a 4-day period. Dark shaded represents nighttime. **(C)** Average of daily food intake of single-housed males EIIARORα WT (white bar, *n* = 7) and EIIARORα KO (black bar, *n* = 7) littermates under high fat diet over 14-week period.

Figure S2 Blood glucose and inflammatory parameters in male EIIARORα WT and EIIARORα KO mice under chow diet. **(A)** Fasted blood glucose level from males EIIARORα WT (white bar, *n* = 7) and EIIARORα KO (black bar, *n* = 7) after 10 weeks of high fat diet (HFD). **(B)** Blood IL-1b level in the males EIIARORα WT (white bar, *n* = 7) and EIIARORα KO (black bar, *n* = 7) after 14 weeks of HFD determined by ELISA. **(C)** WAT gene expression from males EIIARORα WT (white bar, *n* = 7) and EIIARORα KO (black bar, *n* = 7) after 14 weeks of HFD (**p* < 0.05).

Figure S3 **(A)** Fasted blood glucose level from 13-month-old EIIARORα WT males (white bar, *n* = 6) and EIIARORα KO (black bar, *n* = 7) mice fed with normal chow diet. **(B)** Splenocytes from 15-month-old males EIIARORα WT (white bar, *n* = 6) and EIIARORα KO (black bar, *n* = 7) littermates were analyzed by flow cytometry for Th1 [CD3^+^CD4^+^IFNγ^+^ (left panel)], or regulatory T cells (T_reg_) [CD3^+^CD4^+^FoxP3^+^ (right panel)] (**p* < 0.05, ***p* < 0.01, ****p* < 0.001).
