# Supplementary figures and images for: Metabolic Characterization of a Novel RORα Knockout Mouse Model without Ataxia
Source: Front Endocrinol (Lausanne). 2017 Jul 11;8:141. doi: 10.3389/fendo.2017.00141 (PMC5504173; doi:10.3389/fendo.2017.00141)

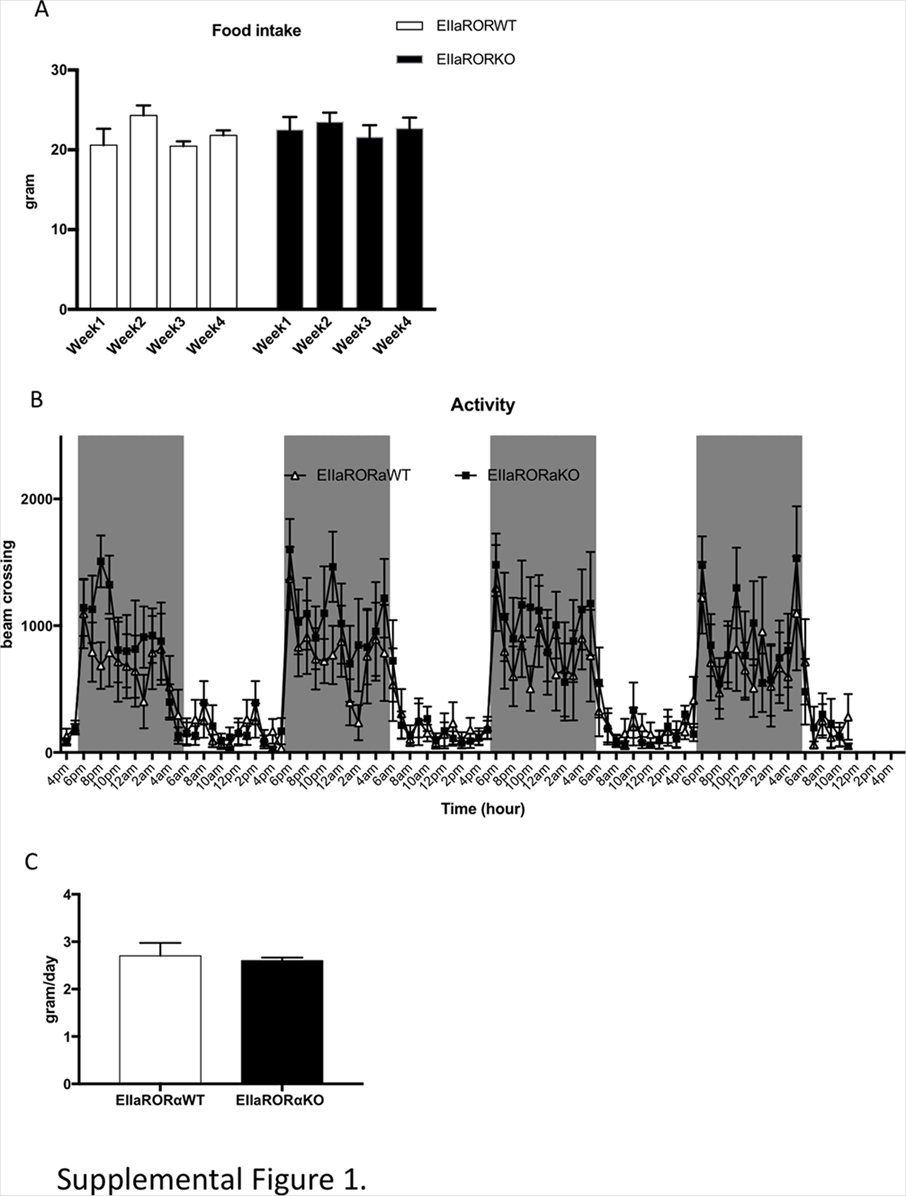

Supplement: Figure S1 — Food intake and activity of single-housed EIIARORα WT and EIIARORα KO mice. (A) Food intake of single-housed 12-week-old males EIIARORα WT (white bar, n = 8) and EIIARORα KO (black bar, n = 8) littermates under chow diet over a month. (B) Recording of single-housed 12-week-old males EIIARORα WT (white triangle, n = 8) and EIIARORα KO (black square, n = 8) littermate activity in metabolic chambers over a 4-day period. Dark shaded represents nighttime. (C) Average of daily food intake of single-housed males EIIARORα WT (white bar, n = 7) and EIIARORα KO (black bar, n = 7) littermates under high fat diet over 14-week period. [file Image_1.tiff]

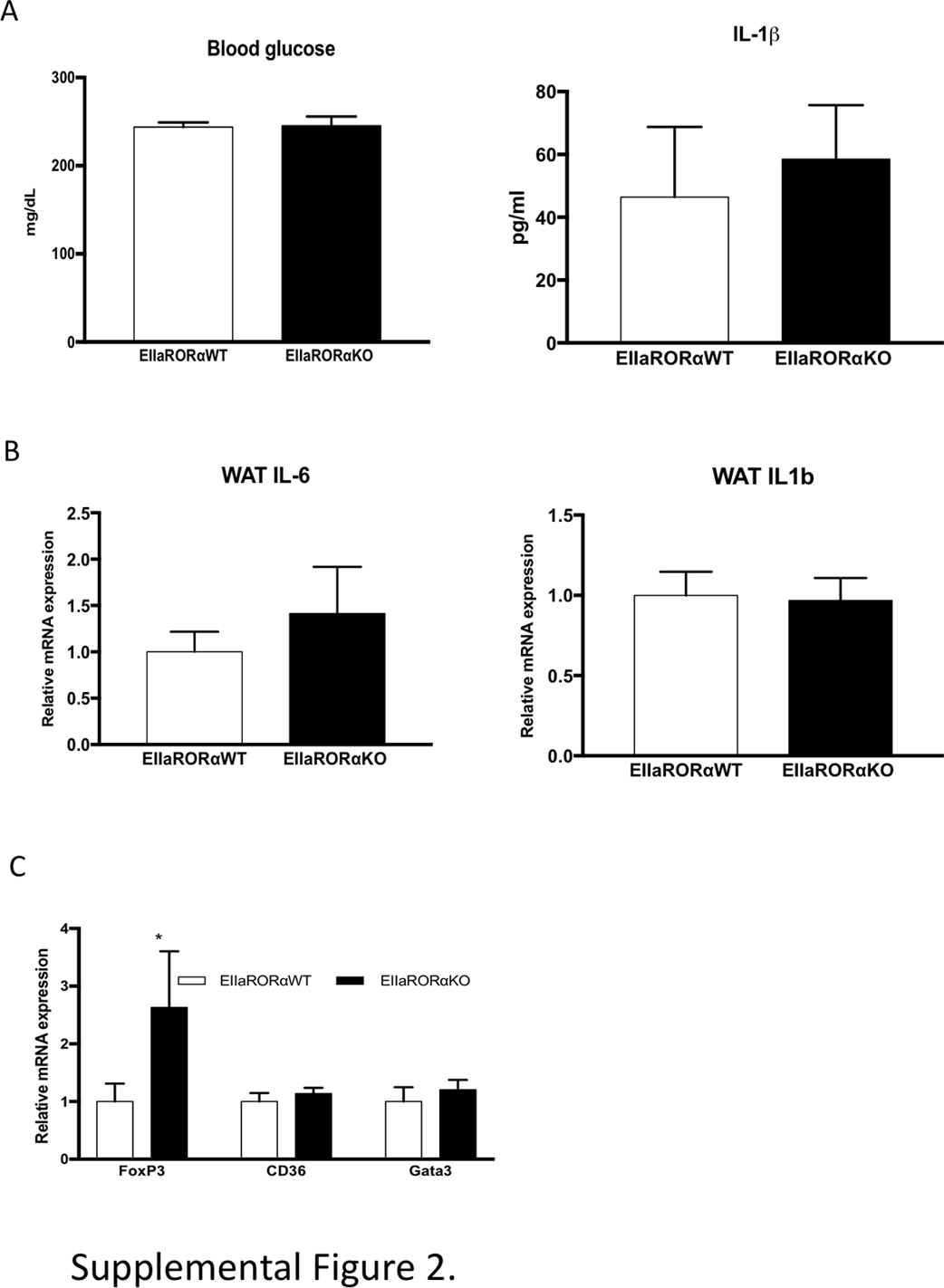

Supplement: Figure S2 — Blood glucose and inflammatory parameters in male EIIARORα WT and EIIARORα KO mice under chow diet. (A) Fasted blood glucose level from males EIIARORα WT (white bar, n = 7) and EIIARORα KO (black bar, n = 7) after 10 weeks of high fat diet (HFD). (B) Blood IL-1b level in the males EIIARORα WT (white bar, n = 7) and EIIARORα KO (black bar, n = 7) after 14 weeks of HFD determined by ELISA. (C) WAT gene expression from males EIIARORα WT (white bar, n = 7) and EIIARORα KO (black bar, n = 7) after 14 weeks of HFD (*p < 0.05). [file Image_2.tiff]

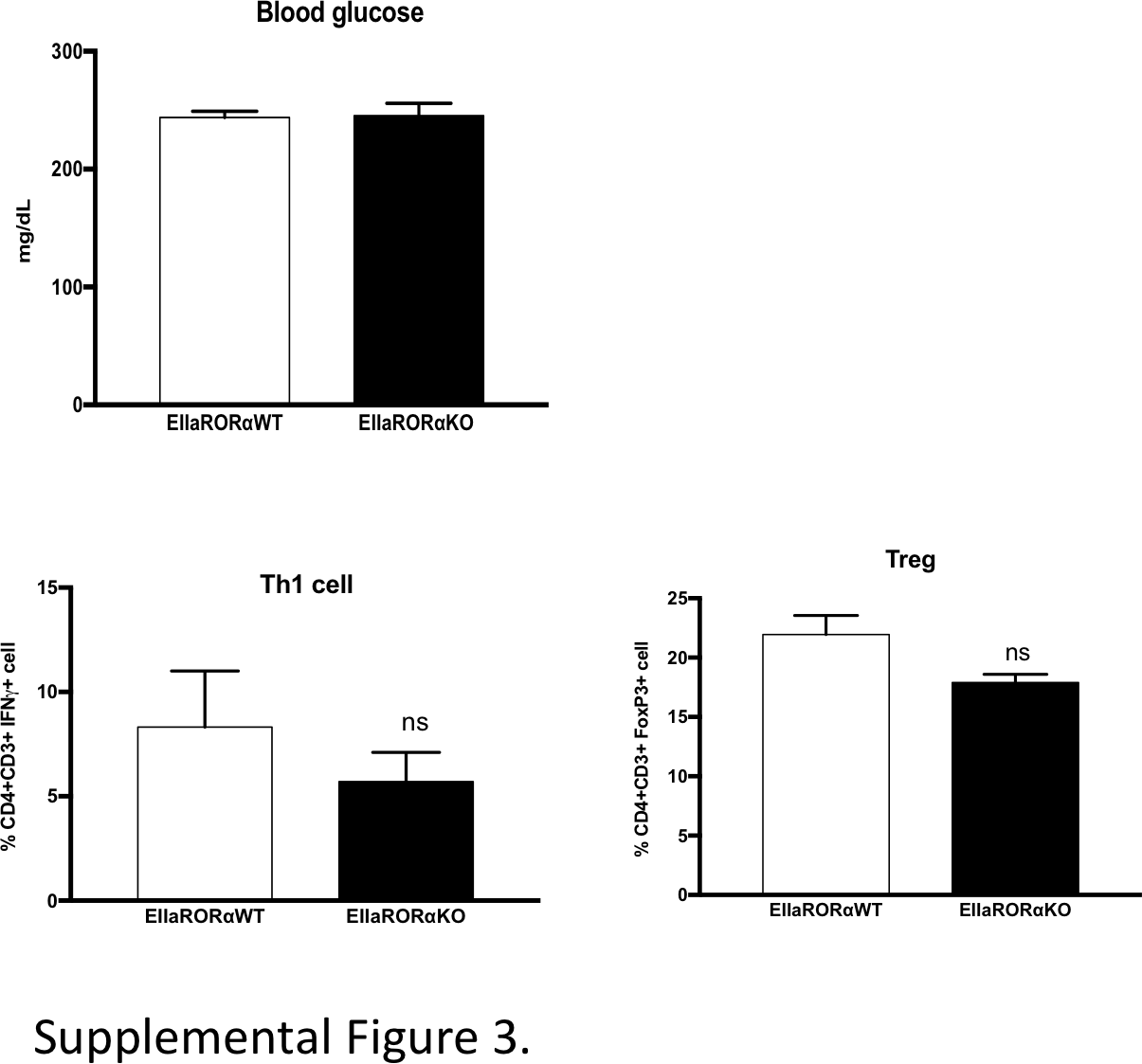

Supplement: Figure S3 — (A) Fasted blood glucose level from 13-month-old EIIARORα WT males (white bar, n = 6) and EIIARORα KO (black bar, n = 7) mice fed with normal chow diet. (B) Splenocytes from 15-month-old males EIIARORα WT (white bar, n = 6) and EIIARORα KO (black bar, n = 7) littermates were analyzed by flow cytometry for Th1 [CD3+CD4+IFNγ+ (left panel)], or regulatory T cells (Treg) [CD3+CD4+FoxP3+ (right panel)] (*p < 0.05, **p < 0.01, ***p < 0.001). [file Image_3.tiff]
