# Supplementary material for: Metabolic Characterization of a Novel RORα Knockout Mouse Model without Ataxia
Source: Front Endocrinol (Lausanne). 2017 Jul 11;8:141. doi: 10.3389/fendo.2017.00141 (PMC5504173; doi:10.3389/fendo.2017.00141)
Supplement: Supplementary file 4 [file Table_1.PDF]

Anti-body	Clone
CD3	17A2
CD4	GK1.5
IL-17R	9B10
Foxp3	MF-14
INFg	XMG1.2
Gata3	16E10A23

Supplemental Table1: Anti-bodies list
