# Supplementary material for: Metabolic Characterization of a Novel RORα Knockout Mouse Model without Ataxia
Source: Front Endocrinol (Lausanne). 2017 Jul 11;8:141. doi: 10.3389/fendo.2017.00141 (PMC5504173; doi:10.3389/fendo.2017.00141)
Supplement: Supplementary file 5 [file Table_2.PDF]

Gene	Forward	Reverse
36B4	ACCTCCTTCTTCCAGGCTT	CCCACCTTGTCTCCAGTCTTT
Cyp7A1	TGGTGGTGAGAGCTTGAAAA	TAGCGAGAGCATGTCGAAAC
Hmgcr	ATGGCTGGGAGCATAGGCGG	CTGCATCCTGGCCACATGCG
Hmgcs	AGGAACGTGGTATCTGGTCA	TGTGTTACTATGCACGAGCC
Sc4mol	TCAGGCTCCATTTGGAATCG	TCCAGCAAACGTATGGTCAC
FASN	GCACAGCTCTGCACTGTCTACTAC	ATCCCAGAGGAAGTCAGATGATAG
SCD1	GGAGACCCCTTAGATCGAGTG	CACTCGAATTACTTCCCACCA
DHCR24	GACGGACGACGTAGAGCCCA	GAGGCCCTCCCGGTTTGTCT
SREBF2	CATCTGCCGGTGGTGGACGT	GCGCACAGCTGCATCGTCTC
Cpt1 alpha	TTGATCAAGAAGTGCCGGACGAGT	GTCCATCATGGCCAGCACAAAGTT
IFN gamma	TGGCTGTTTCTGGCTGTTACT	GCTCTGCAGGATTTTCATGTC
FoxP3	TGGACCACGGGCACTATCACA	GAGGCTGCGTATGATCAGTTATGC
CD36	GCGACATGATTAATGGCACA	CCTGCAAATGTCAGAGGAAA
Cd11c	CCACATTTGTAGAGGCCACC	CTTGGATGCAGAGAAGCTGAC
TGF beta	GCAACAATTCCTGGCGTTAC	GCTGAATCGAAAGCCCTGTA
TNF alpha	TCAGCCGATTTGCTATCTCAT	TGGAAGACTCCTCCCAGGTAT
IL-18	GACTTCACTGTACAACCGCA	AGGCTGTCTTTTGTCAACGA
ROR alpha Del	TTACGTGTGAAGGCTGCAAG	CTGGTCCGATCAATCAAACA
AKT1	GGCTGGCTGCACAAACG	GACTCTCGCTGATCCACATCCT
AKT2	CATAAATAAGGAGCGGGAACGA	GTTTGTGGAGCCAGCCTTCTT
AKT3	CCTTCCAGACAAAAGACCGTTT	CGCTCTCTCGACAAATGGAAA
Glut4	TTGGCTCCCTTCAGTTTGG	CTACCCAGCCACGTTGCAT

Supplemental Table2: qPCR Primers sequences
